# Fractionation of Hydrogen Isotopes by Sulfate- and Nitrate-Reducing Bacteria

**DOI:** 10.3389/fmicb.2016.01166

**Published:** 2016-08-02

**Authors:** Magdalena R. Osburn, Katherine S. Dawson, Marilyn L. Fogel, Alex L. Sessions

**Affiliations:** ^1^Division of Geological and Planetary Sciences, California Institute of TechnologyPasadena, CA, USA; ^2^Life and Environmental Sciences, School of Natural Science, University of California at MercedMerced, CA, USA

**Keywords:** fatty acids, hydrogen isotopes, sulfate-reducing bacteria, anaerobic microbial metabolism, NAD(P)H, transhydrogenase

## Abstract

Hydrogen atoms from water and food are incorporated into biomass during cellular metabolism and biosynthesis, fractionating the isotopes of hydrogen—protium and deuterium—that are recorded in biomolecules. While these fractionations are often relatively constant in plants, large variations in the magnitude of fractionation are observed for many heterotrophic microbes utilizing different central metabolic pathways. The correlation between metabolism and lipid δ^2^H provides a potential basis for reconstructing environmental and ecological parameters, but the calibration dataset has thus far been limited mainly to aerobes. Here we report on the hydrogen isotopic fractionations of lipids produced by nitrate-respiring and sulfate-reducing bacteria. We observe only small differences in fractionation between oxygen- and nitrate-respiring growth conditions, with a typical pattern of variation between substrates that is broadly consistent with previously described trends. In contrast, fractionation by sulfate-reducing bacteria does not vary significantly between different substrates, even when autotrophic and heterotrophic growth conditions are compared. This result is in marked contrast to previously published observations and has significant implications for the interpretation of environmental hydrogen isotope data. We evaluate these trends in light of metabolic gene content of each strain, growth rate, and potential flux and reservoir-size effects of cellular hydrogen, but find no single variable that can account for the differences between nitrate- and sulfate-respiring bacteria. The emerging picture of bacterial hydrogen isotope fractionation is therefore more complex than the simple correspondence between δ^2^H and metabolic pathway previously understood from aerobes. Despite the complexity, the large signals and rich variability of observed lipid δ^2^H suggest much potential as an environmental recorder of metabolism.

## Introduction

The hydrogen isotopic compositions of organic molecules, primarily lipids, have frequently been used as indicators of biosynthetic processes (Chikaraishi and Naraoka, 2001; Chikaraishi et al., 2009), patterns of organic matter cycling (Chikaraishi and Naraoka, 2005; Jones et al., 2008), and as proxies for the isotopic composition of water in paleoenvironmental reconstructions (Sauer et al., 2001; Huang et al., 2002; Sachse et al., 2012). To first order, the hydrogen isotopic composition (expressed as a δ^2^H-value) of organic matter reflects that of growth water, offset by some (often large) fractionation associated with biosynthesis (Estep and Hoering, 1980; Sessions et al., 1999; Hayes, 2001). However, very large ranges of δ^2^H-values are observed in natural samples, even those occurring in a single species or environment, indicating that other processes are important as well (Jones et al., 2008; Li et al., 2009; Osburn et al., 2011). Known sources of variability include environmental conditions that may change the isotopic composition of intracellular water, such as salinity in planktonic organisms and aquatic macrophytes or humidity and transpiration in plants (Sachse and Sachs, 2008; Pu and Weiguo, 2011; Romero-Viana et al., 2013), or changes in biosynthetic fractionation such as between isoprenoid and *n*-alkyl lipids (Sessions et al., 1999). In addition, it has been shown that the central metabolic pathways used by microorganisms to process organic substrates are correlated with lipid δ^2^H (Zhang et al., 2009). These metabolism-associated isotope effects span up to a 500‰; range for a specific fatty acid in a single microbial strain, far exceeding most other forms of variability.

Patterns of lipid δ^2^H proposed by Zhang et al. (2009) include strong ^2^H-enrichment of lipids in aerobic heterotrophs, strong ^2^H-depletion in chemoautotrophs, and intermediate ^2^H-depletion by photoautotrophs. During heterotrophic growth, the level of ^2^H-enrichment is loosely correlated with the central metabolic pathway being activated by a particular substrate, with growth on tricarboxylic acid (TCA) cycle intermediates like acetate and succinate producing the most extreme ^2^H-enrichments (Zhang et al., 2009). Such trends have subsequently been replicated in other (aerobic) bacterial strains (Heinzelmann et al., 2015a,b), an archaeon (Dirghangi and Pagani, 2013b), and a heterotrophic eukaryote (Dirghangi and Pagani, 2013a). Isotope effects associated with the enzymatic protonation (reduction) of NADP^+^ during central metabolism, which can lead to either ^2^H depletions or enrichments in NADPH (Zhang et al., 2009), have been hypothesized to explain metabolic trends in hydrogen isotopes. This hydrogen is then transferred to lipids as an intact hydride donor in biosynthetic reactions (White, 2000; Zhang et al., 2009), facilitating transfer of the isotope label. Several recent culturing studies support this hypothesis (Dirghangi and Pagani, 2013b; Dawson et al., 2015; Heinzelmann et al., 2015a,b).

Using hydrogen isotopic fractionations as a proxy for microbial metabolism has considerable potential utility as an environmental indicator, extending the role of compound specific lipid δ^2^H beyond recording environmental water, to recording metabolic diversity. Because lipids have far greater preservation potential than nucleic acids, they may provide a coarser bur much longer-lasting record than do genomic approaches. Evaluation of these trends in modern environmental samples including hot springs (Naraoka et al., 2010; Osburn et al., 2011), marine particulate organic carbon (POC; Jones et al., 2008), and marine sediments (Li et al., 2009; Heinzelmann et al., 2015c) have revealed large ranges in lipid δ^2^H that are potentially attributable to heterotrophic ^2^H-enrichment and chemoautotrophic ^2^H-depletion. In addition, recent culturing studies have shown the metabolic effects to be larger in magnitude than those stemming from growth phase or salinity (Dirghangi and Pagani, 2013a,b; Chivall et al., 2014; Dawson et al., 2015; Heinzelmann et al., 2015a,b).

While these initial studies are promising, they include data primarily from aerobic heterotrophs and chemoautotrophs (Zhang et al., 2009; Heinzelmann et al., 2015b). Microbes with anaerobic metabolism are widespread in both marine and terrestrial environments and are often of great interest in reconstructing paleoenvironmental and paleoecological conditions. To date, published data for anaerobic growth includes the following: three photoheterotrophs (Zhang et al., 2009; Heinzelmann et al., 2015b), three sulfate reducers (Campbell et al., 2009; Dawson et al., 2015; Leavitt et al., 2016), and an acetogen (Valentine et al., 2004), primarily phototrophic or chemoautotrophic microbes. Dawson et al. (2015) analyzed the anaerobic heterotroph *Desulfococcus multivorans* and measured an 80‰; range in hydrogen isotope fractionation between lipids and growth water (ε_L–W_) for pure cultures grown on a range of heterotrophic substrates. The growth substrates that lead to the most ^2^H-enriched lipids were acetate and succinate, consistent with previous findings; however, ε_L–W_ averaged −137 and −127‰;, significantly ^2^H-depleted relative to analogous aerobic cultures. This discrepancy, along with the general lack of published information on hydrogen isotope fractionations associated with anaerobic heterotrophs, arguably a dominant metabolism in sedimentary environments worldwide, is the primary motivation for our study.

Here we report on hydrogen isotopic fractionations in lipids produced by several facultative and obligate anaerobes. The organisms chosen for this study were selected in order to make direct comparisons of autotrophic vs. heterotrophic growth under both aerobic and anaerobic conditions. *Escherichia coli, Paracoccus denitrificans, and Shewanella oneidensis* can be grown under nearly identical conditions with either O_2_ or nitrate as an electron acceptor, providing a direct comparison between aerobic and anaerobic growth. *P. denitrificans* can also grow chemoautotrophically. We also studied five strains of strictly anaerobic sulfate reducing bacteria (SRB), all *Deltaproteobacteria* but differing significantly in both their specific metabolic function and known biochemical pathways (Muyzer and Stams, 2008). They include representatives with very broad heterotrophic specificity (*D. autotrophicum, D. propionicus*, and *D. multivorans*) as well as those capable of both autotrophic and heterotrophic growth (*D. autotrophicum, D. hydrogenophilus*, and *D. multivorans*). *D. alaskensis* provides a direct point of comparison to the recent work of Leavitt et al. (2016). Our results suggest that significant differences in ε_L–W_ exist between nitrate-reducing and sulfate-reducing organisms. Whether such patterns are widespread among anaerobes remains to be determined.

## Materials and methods

### Bacterial strains and culturing conditions

Strains for growth trials were selected based on their metabolic capability, sequenced genomes, and accessibility. Five SRBs, *Desulfobacterium autotrophicum* (DSM 3382), *Desulfobacter hydrogenophilus* (DSM-3380), *Desulfobulbus propionicus* (DSM 2032), *Desulfovibrio alaskensis* (DSM 16109), and *D. multivorans* (DSM 2059) were purchased from the DSMZ culture collection. *E. coli* K-12 MG1655 is the same strain as in Zhang et al. (2009). *Paracoccus denitrificans* (B-3785) was supplied by the ARS culture collection. The laboratory of Kenneth Nealson at the University of Southern California supplied *S. oneidensis*.

Strains were all grown in minimal media with single carbon sources (Table 1). Media were made up in deionized water (Milli-Q system), and where required the medium δ^2^H-value was adjusted by addition of filter-sterilized ^2^H_2_O. *P. denitrificans* and *E. coli* were grown in a low salt base (LWB) for all experiments. LWB contained per liter of water: 0.1 g NaCl, 0.5 g MgSO_4_·7H_2_O, 0.1 g CaCl_2_·2H_2_O, 0.05 g KCl, 0.03 g NH_4_Cl, 0.11 g KH_2_PO_4_·H_2_O, 1 ml vitamin solution, and 1 ml trace element solution (Breznak and Leadbetter, 2006). Sulfate-reducers were grown in Medium 383 (DSMZ) or Medium 194 (DSMZ; *D. propionicus*) supplementing the basal media with the trace element solution from the LWB medium, buffering with 1 g/L NaHCO_3_, and heterotrophic carbon source to 10 mM. Anaerobic media was cooled and prepared in an anaerobic chamber prior to flushing of the headspace with 80:20 N_2_:CO_2_ or H_2_:CO_2_ if H_2_ was the electron donor. Aerobic experiments were loosely capped and open to the atmosphere with the exception of cultures requiring H_2_ and O_2_, where media was degased under H_2_:CO_2_ and lab air was introduced by filter sterilization. Sulfate reducers were grown without shaking in the dark, whereas facultative anaerobes were shaken at ~120 RPM. Flasks, Balch tubes, and serum bottles for growth experiments were baked in a muffle furnace at 475°C prior to use. Cultures for lipid collection (generally 100 ml) were inoculated with 0.5–1 ml of actively growing culture before incubation at 25°C (*E. coli, P. denitrificans*, and *S. oneidensis*) or 28°C (SRBs). Growth curves were produced for each condition by monitoring optical density at 600 nm in separate smaller volumes with identical media with growth rates and doubling time calculated from regression of the exponential phase of growth curves after Widdel (2010). Cultures were harvested in late exponential or early stationary phase. Biological duplicates were produced for most growth conditions as indicated in Table 1 by multiple water isotope measurements.

**Table 1:** **Culture conditions**.

**Sample name**	**e-Donor**	**e-Acceptor**	**Generation time (h)**	**δ^2^H_water_**
***Paracoccus denitrificans***				
PD_A	Acetate	O_2_	2.3	−69, −65, −57, 292, 430
PD_An	Acetate	NO_3_	32.4	−67, −49
PD_S	Succinate	O_2_	2.1	−69, −69, −66, 265, 394, −85, 177, 421, 635, 910, 1180
PD_Sn	Succinate	NO_3_	66.9	−71, −67, 263, 394, 194, 437, 691, 976, 1181, 1409
PD_P	Pyruvate	O_2_	2.9	−68, −55
PD_Pn	Pyruvate	NO_3_	25.7	−76, −67
PD_L	Lactate	O_2_	2.4	−82, −73, −53
PD_Ln	Lactate	NO_3_	65.4	−82, −67
PD_G	Glucose	O_2_	2.1	−69, −65, 288, 433
PD_Gn	Glucose	NO_3_	23.5	−67, −60
PD_T	Thiosulfate	O_2_	14.5, 13	−82, −29.5, 11, 51, 91
PD_Tn	Thiosulfate	NO_3_	17.3	−83
PD_M	MeOH	O_2_	1.2	−38
PD_H	H_2_	O_2_	4.9	−82, −64, 3, 209
PD_Hn	H_2_	NO_3_	25.7	−69, −64, −41, 289, 427
***Escherichia coli***				
EC_A	Acetate	O_2_	12.8	−82, −62
EC_An	Acetate	NO_3_	12.4	−80, −67, −13
EC_S	Succinate	O_2_	3.6	−83, −60
EC_Sn	Succinate	NO_3_	3.9	−79, −67
EC_P	Pyruvate	O_2_	3.7	−82, −65
EC_Pn	Pyruvate	NO_3_	7.7	−77, −69, −2.6
EC_L	Lactate	O_2_	3.8	−87, −63
EC_Ln	Lactate	NO_3_	3.2	−83, −67
EC_G	Glucose	O_2_	1.6	−82, −55
EC_Gn	Glucose	NO_3_	4.3	−79, −70, 43, 170
EC_Gf	Glucose	−	4.7	−79, −70
***Shewanella oneidensis***				
SO_L	Lactate	O_2_	X	−58
SO_Ln	Lactate	NO_3_	X	−66
SO_Lf	Lactate	fumarate	X	−65
***Desulfobacterium autotrophicum***				
DA_A	Acetate	SO_4_	43.0	−70, −70
DA_S	Succinate	SO_4_	43.6	−66, −67
DA_F	Formate	SO_4_	56.8	−75, −71, −70
DA_P	Pyruvate	SO_4_	34.2	−75, −71, −70
DA_G	Glucose	SO_4_	73.4	−68, −68
DA_L	Lactose	SO_4_	X	−75
DA_H	H_2_	SO_4_	33.8	−70, −64
***Desulfobacter hydrogenophilus***				
DH_A	Acetate	SO_4_	20.7	−76, −67
DH_H	H_2_	SO_4_	32.3	−71, −60
***Desulfovibrio alaskensis***				
Dv_P	Pyruvate	SO_4_	7.8	−73, −68, 57, 176
Dv_L	Lactate	SO_4_	5.9	−76, −67, 71, 186
***Desulfobulbus propionicus***				
DB_A	Acetate, H_2_	SO_4_	51.0	−76
DB_L	Lactate	SO_4_	20.6	−68, −70
DB_P	Pyruvate	SO_4_	15.4	−71, −70
DB_E	Ethanol	SO_4_	18.7	−73, −70
Desulfococcus multivorans^**^				
DM_A	Acetate	SO_4_	144.9	−72
DM_S	Succinate	SO_4_	105.5	−66
DM_P	Pyruvate	SO_4_	90.3	−65
DM_L	Lactate	SO_4_	68.6	−71
DM_G	Glucose	SO_4_	145.1	−60
DM_B	Benzoate	SO_4_	107.9	−72
DM_F	Formate	SO_4_	92.2	−63

*X, not measured*.

** *From Dawson et al. (2015)*.

### Lipid extraction and identification

Harvested cultures were pelleted by centrifugation, frozen, lyophilized, and stored at −20°C until lipid extraction. Fatty acid methyl esters (FAMEs) were produced from cellular material in a combined extraction/derivatization procedure based on Rodríguez-Ruiz et al. (1998). For each sample, 1–100 mg of dry biomass was combined with 1 ml of hexane and 2 ml of 20:1 anhydrous methanol: acetyl chloride and reacted in a sealed glass culture tube at 100°C for 10 min. After cooling, 2 ml of water was added to the mixture followed by 3-fold extraction with 3 ml hexane. Where necessary, separation of saturated from unsaturated FAMEs was undertaken using Discovery®Ag-Ion solid phase extraction columns (Supelco), eluting the saturated, monounsaturated, and diunsaturated FAMEs in 96:4 hexane: acetone (6 ml), 90:10 hexane: acetone (4 ml), and acetone (4 ml), respectively. Fractions were evaporated to dryness under N_2_ and suspended in 1 ml hexane for analysis.

Fatty acids were identified by gas chromatography–mass spectrometry (GC-MS) and quantified via flame ionization detector (GC-FID). One microliter of each organic extract was injected into a ThermoFinnigan Trace GC with the effluent split ~9:1 between a DSQ mass spectrometer and FID. The sample was injected into a programmable temperature vaporization (PTV) injector operated in splitless mode and heated to 330°C in 24 s. The GC was equipped with a ZB-5 ms capillary column (30 m long, 0.25 mm I.D., 0.25 μm film) and was operated with a He carrier gas flow rate of 0.8 ml/min. The oven temperature was held for 1 min at 100°C, ramped at 20°C/min to 140°C, ramped at 3.0°C/min to 250°C and held for 1 min, then ramped at 20°C/min to 310°C and held for 10 min. Compounds were identified by comparison of mass spectra to the NIST 2004 library and/or by retention time to authentic standards. Samples were quantified relative to a known amount of either palmitic acid isobutyl ester (PAIBE) or a C_23_ *n*-alkane internal standard. The position and stereochemistry of double bonds was not determined.

### Isotopic analyses

Samples of culture medium for water hydrogen isotope analysis were collected either prior to incubation, or post-incubation by filtering through a 0.2 μm syringe filter or by collection of centrifuge supernatant after pelleting cells. Waters were analyzed using a Los Gatos DLT-100 spectroscopic Liquid Water Isotope Analyzer in eight-fold replicate against appropriate working standards (δ^2^H = −117.0‰;, −10.6, +287.3, and +457.6). Spectral interference was noted only for cultures containing MeOH, for which measurements of isotopic composition were made prior to adding the carbon substrate. Measured isotope ratios were converted to δ^2^H-values by comparison with the four standards, and normalized to the VSMOW-SLAP scale. Typical precision for these analyses was 1–2‰. All data reduction was performed using Visual Basic code written by us.

δ^2^H-values of FAMEs were measured using a ThermoFinnigan Trace GC coupled to a Delta+XP isotope ratio mass spectrometer (IRMS) via a pyrolysis interface (GC/TC) operated at 1391°C. External FAME standards were analyzed after every four samples. Eight to twenty-four microliters of each sample was injected using a PTV injector operated in splitless mode with solvent venting. A thick-film ZB-5 ms column (30 m long, 0.25 mm I.D., 1.00 μm film) was employed with He carrier gas flow rate at 1.4 ml/min. The GC oven temperature was held at 100°C for 1 min, ramped at 20°C/min to 205°C, ramped at 0.8°C/min to 220°C, ramped at 8°C/min to 320°C and held for 10 min. Peaks were identified by comparison of retention order and relative height to GC-MS chromatograms. Isotope ratios were calculated using ISODAT NT 2.5 software by comparison to methane reference gas peaks as described previously (Wang and Sessions, 2008) and are reported in the conventional δ^2^H notation (≡ R_samp_/R_std_ −1) as permil (‰) deviations from the VSMOW standard. The root-mean-squared (RMS) error for external FAME standards run both before and between sample runs was 3.28 ± 0.8‰ (*n* = 190). Data were corrected for the calculated offset of bracketing external FAME standards. The standard deviation for replicate analyses of unknown analytes averaged 7.5‰ (*n* = 247). Samples were analyzed in triplicate. The ${\text{H}}_{3}^{+}$ factor averaged 3.48 ± 0.16 ppm/mV. Lipid isotope measurements were also corrected to exclude hydrogen contributed by the derivative methyl group. This was accomplished through determination of the MeOH δ^2^H by derivatizing a phthalic acid of known non-exchangable δ^2^H, then calculating non-exchangable lipid δ^2^H by isotopic mass balance. Fractionations between lipids and environmental water were calculated as ε_L–W_ = ((δ^2^H_*l*_+1)/(δ^2^H_w_+1) − 1) and are reported as permil (‰) variations.

Bulk biomass δ^2^H-values were measured in a Thermo Fisher thermo-chemolysis/elemental analyzer (TC/EA) interfaced through a Conflo IV into a Delta V Plus isotope ratio mass spectrometer at the University of California, Merced. Briefly, 0.1–0.4 mg of dried samples were weighed in duplicate into 3 × 5 mm silver capsules, sealed, then shipped to Merced, CA. Samples were held in ambient air for >1 month prior to final drying in a desiccator at room temperature. Silver boats with samples and standards were loaded into a Zero-blank autosampler (Costech Analytical) and purged with zero grade He for 10 min. Samples were introduced into a graphite and glassy carbon furnace at 1450°C. H_2_ was separated from CO via molecular sieve chromatography. The δ^2^H-values were determined relative to five standard compounds analyzed by the same methodology: stearic acid, mineral oil (Isoanalytical), pump oil (Estep and Hoering, 1980), ground chicken feather (Chris Romanek), and ground turkey feather (Chris Romanek). Stearic acid, mineral oil, and pump oil have hydrogen in non-exchangeable positions, whereas the chicken and turkey feathers potentially can exchange about 10% of their H at room temperature in air. Duplicate δ^2^H analysis of samples were typically within ±2‰ of an average value. These measurements include a small (~10%) portion of theoretically exchangeable hydrogen at room temperature. Because samples were of similar chemical composition, allowed to equilibrate with ambient atmospheric water vapor, and handled identically, the error associated with such exchangeable H is small. Two of the standards used (turkey and chicken feather) have similar percentages of “exchangeable hydrogen.” Part of our data processing includes comparing our measurements with δ^2^H-values of theoretically non-exchangeable hydrogen.

## Results

**Figure 1:**
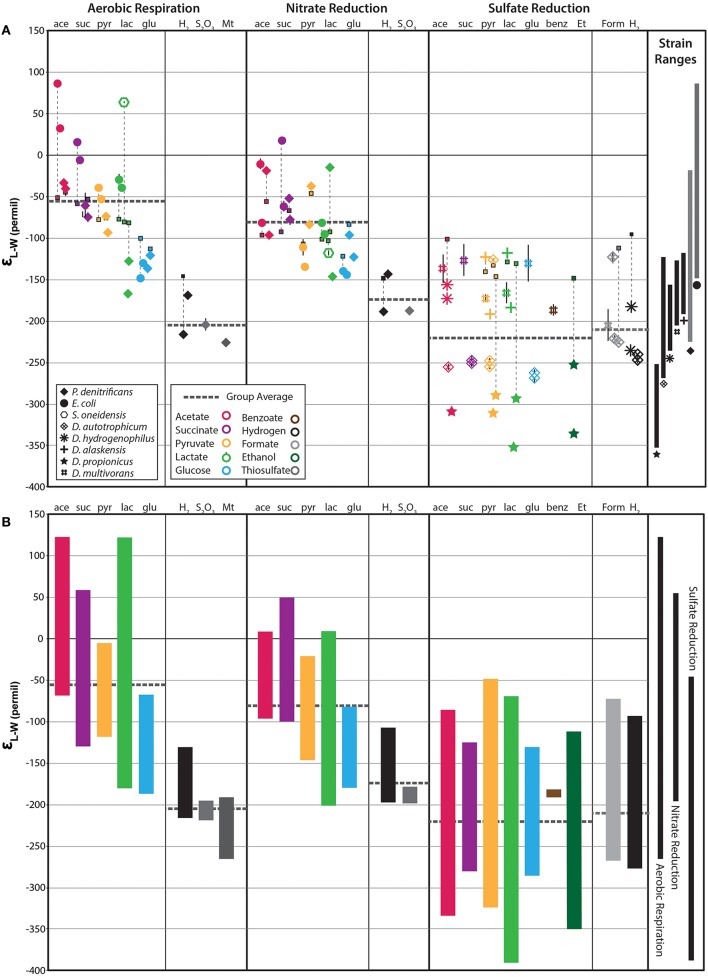
**Fractionations between fatty acids and water (ε_**L−W**_) for all non-labeled experiments**. **(A)** Individual symbols represent the abundance-weighted average ε_L−W_ for all fatty acids measured in a given culture experiment. Bulk biomass δ^2^H-values are shown as small squares with thin vertical dashed lines extending to the corresponding **ε**_L−W_-value. Thick, horizontal, dark gray, dashed lines indicate the group average (e.g., heterotrophic aerobic respiration). The range of abundance-weighted average **ε**_L−W_ for each strain is shown on the far right. **(B)** Vertical colored bars indicate the total compound-specific range for each substrate condition. Compound-specific data is plotted in Figure S1. Black bars on the far right illustrate the total range of compound specific data for aerobic respiration, nitrate reduction, and sulfate reduction, respectively.

Each strain produced a characteristic suite of fatty acids (FA) whose identity and abundance varied substantially between strains, but were generally consistent in the same strain grown on different substrates (see Supplementary Table). The SRBs produced a more complex mixture of fatty acids often including branched and odd chain FA whereas *E. coli, P. denitrificans, and S. oneidensis* produced a more limited set (Figure S1), consistent with previous reports (Taylor and Parkes, 1983; Dowling et al., 1986). The range of δ^2^H-values measured for different FA structures from a single culture was often large, particularly for the SRB (Figure 1B and Figure S1). For example, *D. autotrophicum* grown autotrophically on H_2_ yielded a range of fatty acid δ^2^H-values from −210 to −315‰ (for 18:0 and 16:1 FA, respectively). This range shifted to −217 to −325‰ (for 16:0 and cyc-16:0 FA, respectively) when grown on succinate. The δ^2^H-values and relative ordering of individual compounds varied considerably across both substrates and strains. While there are tendencies for some structures (e.g., branched FA) to be relatively enriched or depleted, no robust inter- or intra-strain trends are apparent in this dataset (Figure S1). To allow for quantitative comparison between strains that produce different fatty acids, we calculated the abundance-weighted mean fractionation between lipids and water (ε_L–W_) using all measured fatty acids (Figure 1A). Weighted-mean epsilon values for the *D. autotrophicum* trials discussed above are −247 and −250‰, respectively, indicating that the change in substrate does not significantly alter the isotopic composition of the overall fatty acid pool. In contrast, ε_L–*W*16:0_ values are −235 and −278‰; for the two growth conditions, respectively. These results demonstrate the dynamic and complex nature of hydrogen isotope fractionation, particularly if individual molecular structures are considered in isolation. A summary of the observed fractionations for each organism, grouped by growth condition and substrate, is presented in Figure 1.

We observe a large range of ε_L–W_-values that vary between both strains and growth substrates. The fractionations exhibited by aerobes (left-hand group in Figure 1) vary strongly with substrate, with each strain exhibiting a large range of values (~250‰). The same organisms grown anaerobically under nitrate-reducing conditions (middle panel of Figure 1) yielded a generally similar, albeit slightly diminished, pattern of fractionation. Growth on lactate was a significant exception (see below). In contrast, fractionations measured for SRBs (right-hand panels of Figure 1) did not vary significantly between growth substrates, but did vary substantially between individual strains.

### O_2_ respiring cultures

Cultures grown under aerobic conditions produced the broadest range of mean fractionation, ranging from −226 to +86‰ (average −80‰; Figure 1). The previously observed correspondence with metabolic substrate is apparent, with significant ^2^H-enrichments observed for lipids under heterotrophic conditions (−167 to +86‰, average −56‰) and ^2^H-depletions for autotrophic conditions (−226 to −169‰, average −204‰). Moreover, growth on acetate yielded the most positive values for ε_L–W_ (−40 to +80‰), followed by succinate (−74 to 15‰), pyruvate (−93 to −39‰), and glucose (−148 to −121‰), the same order as has previously been observed (Zhang et al., 2009). Cultures grown on lactate produced a very large range of fractionations (−167 to +63‰), with the four individual strains varying systematically within this range. For example, lipids from *P. denitrificans* cultures grown on lactate are relatively ^2^H-depleted (lower ε_L–W_) and are similar to those grown on glucose, whereas lipids from *E. coli* cultures are more ^2^H-enriched (higher ε_L–W_). *S. oneidensis* produced very ^2^H-enriched lipids while growing aerobically on lactate.

**Figure 2:**
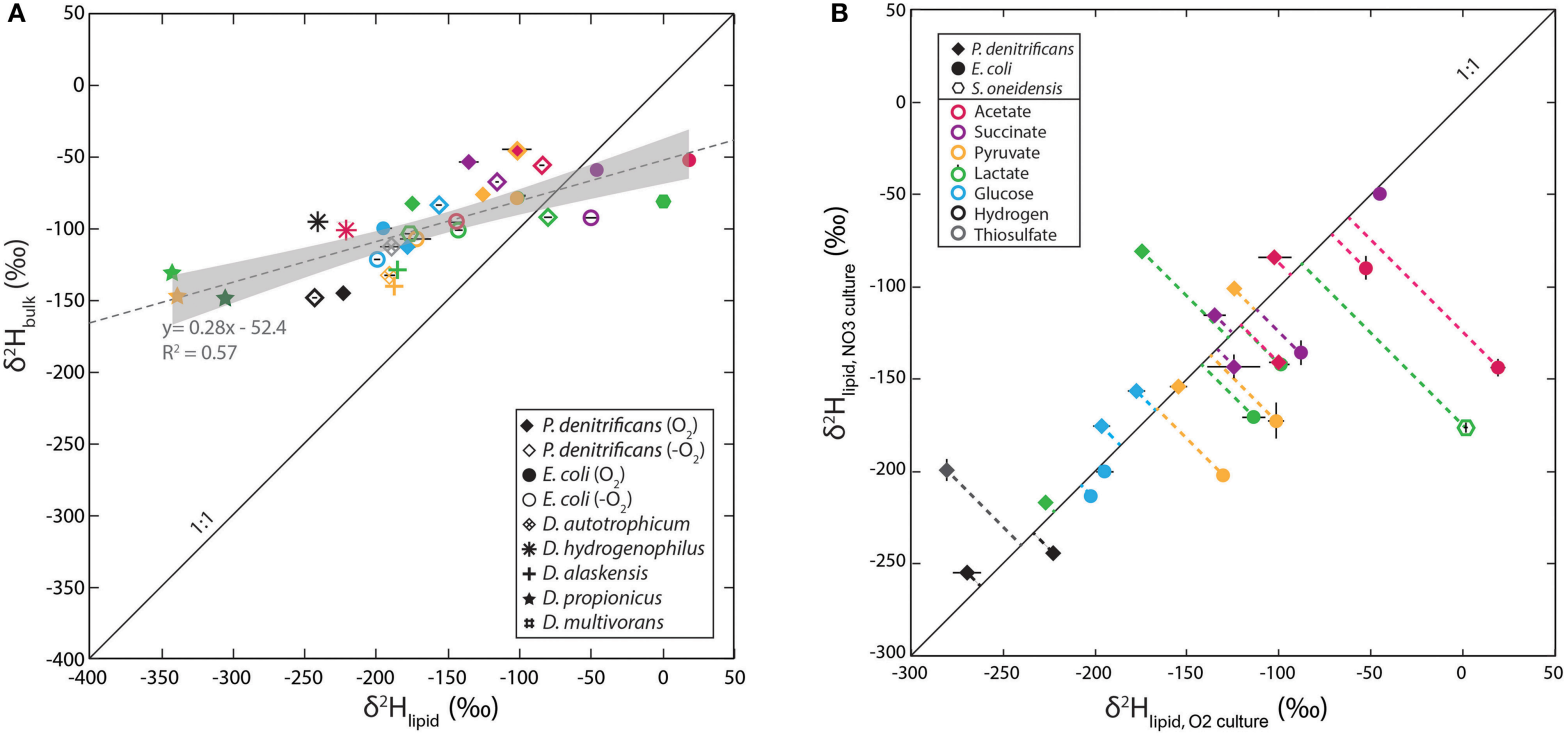
**(A)** Cross-plot of lipid vs. bulk cellular δ^2^H-values. The dotted gray line in (A) is a linear regression of the data and gray shaded envelope shows 95% confidence intervals on the regression. **(B)** Cross-plot of δ^2^H-values for aerobic and anaerobic culture conditions for facultative anaerobes. Symbols reflect strains type and color reflects substrate in both graphs. Solid diagonal lines are slopes of 1:1 plotted for reference.

Lipid δ^2^H and bulk cellular biomass δ^2^H-values are strongly correlated, as would be expected (Figure 2A). However, the range of values in bulk δ^2^H (−50 to −150‰) is only about half that of the abundance-weighted mean lipid values (−80 to +100‰), with the two crossing at around −100‰. The greater range of lipid δ^2^H relative to that of bulk biomass cannot be attributed solely to exchangeable hydrogen, as this would require >50% exchangeable H. Possible origins of this pattern are discussed below in Section NADPH Isotope Exchange and Cellular Flux Effects.

### Nitrate reducing cultures

Lipid-water fractionations observed for nitrate-reducing cultures were—with a few exceptions—broadly similar to those from aerobic cultures. The total range of ε_L–W_-values for these cultures was −147 to + 18‰, averaging −81‰, which is 25‰ more ^2^H-depleted than the average for aerobic heterotrophy (dashed horizontal lines, Figure 1). Autotrophic nitrate-reducing cultures ranged from −188 to −143‰, averaging −173‰, 31‰ more ^2^H-enriched than their aerobic equivalents. However, neither of the paired differences between aerobic and anaerobic growth are significantly different than zero (*p*-values are 0.18 and 0.19, respectively). Figure 2B compares fractionation data between analogous experiments (same organism, substrate) respiring O_2_ vs. nitrate. Symbols plotting to the right of the diagonal are ^2^H-enriched in the aerobic condition whereas those plotting to the left are ^2^H-enriched in the anaerobic condition. Even at the level of individual substrates, there is no systematic pattern of variation between aerobic and anaerobic growth. For instance, acetate and lactate respiration yield lipid ^2^H-enrichment in the aerobic condition for *E. coli* and *S. oneidensis* but not *P. denitrificans*. Glucose respiration yields similar fractionations under both conditions for all strains, whereas H_2_ oxidation yields slight lipid ^2^H-enrichment for the anaerobic condition. The relationship between bulk biomass and lipid δ^2^H-values for nitrate reducing cultures is broadly similar to that observed in aerobes (Figure 2A).

### Sulfate reducing cultures

In contrast to the cultures of aerobic and nitrate-respiring bacteria, fractionations for the sulfate-reducing cultures seemed to differ mainly between strains, rather than between growth substrates (Figure 1). The total range in in ε_L–W_ observed for heterotrophic sulfate-reducers is −352 to −117‰, but the combined strain average ε_L–W_ for acetate, succinate, pyruvate, lactate and glucose are all similar (−214, −208, −214, −222, and −220‰, respectively). The observed difference between the population mean ε_L–W_-values of sulfate reducers and facultative anaerobes (O_2_ and NO_3_ respiring cultures) has strong statistical support (*p* < < 0.0001, student *t*-test) indicating that hydrogen isotopic fractionation patterns in lipids are controlled by different processes. The most divergent strain is *D. propionicus* whose entire range of ε_L–W_-values are more ^2^H-depleted than those of the facultative anaerobes and only overlaps in one case with the next most ^2^H-depleted SRB *D. autotrophicum*. Notably, the lipids of the heterotrophic SRB are not particularly ^2^H-enriched, instead covering much of the range attributed previously to chemolithoautotrophs (Zhang et al., 2009; Heinzelmann et al., 2015b). In addition, the range of lipid ε_L–W_-values for autotrophic sulfate reducers (including C1 formate oxidation) is −247 to −123‰, averaging −210‰, slightly ^2^H-enriched relative to the heterotrophic SRB cultures. These ranges include the data from Dawson et al. (2015) for *D. multivorans* (Figure 1, # symbols). Data from these cultures are generally similar to lipid ε_L–W_-values for *D. hydrogenophilus* and *D. alaskensis*, but enriched relative to the lipid ε_L–W_-values of *D. propionicus* and *D. autotrophicum*.

## Discussion

Our data show that the δ^2^H-values of lipids can vary between strain, growth substrate (or electron donor), and electron acceptor. MANOVA (multivariate analysis of variance) tests on the whole data set reveal that strain (*p* = 0.0074) and electron acceptor (*p* = 0.02281) are significant in describing variation in ε_L–W_ but substrate is not (*p* = 0.956). The importance of electron acceptors in influencing lipid δ^2^H had not previously been appreciated. Differences in fractionation for both *E. coli* and *P. denitrificans* when respiring O_2_ vs. nitrate were relatively small. However, it is not obvious that they should differ at all, given that the relevant respiratory chains are nearly identical until the terminal reduction step and that nitrate anion carries no hydrogen atoms. Large differences in fractionation by sulfate reducers are perhaps less surprising, given that they are metabolically quite distinct. Nevertheless, SRB also conserve reducing equivalents (i.e., NADPH) via many of the same pathways as other bacteria, including the pentose phosphate pathway, TCA cycle, and transhydrogenases, so we expected fractionation to vary with growth substrate, which it did not. The lack of isotopic variation assigned to substrate-dependent fractionation in these organisms is perhaps an important clue toward understanding why ε_L–W_ *is* substrate dependent in aerobes and nitrate-reducers. In any event, a mechanistic understanding of these trends is critical to the future use of lipid δ^2^H as a recorder of microbial metabolism. Next we evaluate our data in light of known genetic components of each of organism and their measured growth rates, then explore a number of additional mechanisms that could contribute to such variability, highlighting key experiments that may help resolve this question in the future.

### Central metabolic pathways

Current ideas about lipid δ^2^H variability assume that organic substrates provide only a small (<25%) proportion of lipid hydrogen, thus changes in the δ^2^H-values of substrates alone cannot explain the large changes observed in lipids (Zhang et al., 2009), although they may contribute to it. In contrast, more than half of FA hydrogen is attributed to direct reductions by NADPH, which is in turn produced during central carbon metabolism (Sessions et al., 1999; Zhang et al., 2009; Dirghangi and Pagani, 2013a,b; Heinzelmann et al., 2015b) with hydrogen ultimately derived from water. Moreover, the isotopic composition of growth substrates generally cannot be measured in natural environments. Thus, the measured fractionation between lipids and growth water serves as a convenient, albeit incomplete, description of the relevant cellular fractionations that we seek to understand. In the following discussion, we therefore focus on trying to explain values of ε_L–W_.

Current data suggest modest ^2^H-enrichments of bacteria utilizing substrates that are processed within and through the TCA cycle (acetate, succinate, malate), modest ^2^H-depletion for those using glycolytic and pentose phosphate pathways, and significant ^2^H-depletions for those using chemoautotrophic metabolisms. Such patterns have generally been attributed to isotope effects accompanying the reduction of NADP^+^ by various enzymes (Zhang et al., 2009). One possible difference between aerobes and SRB is thus their compliment of NADP-reducing enzymes. As noted by Leavitt et al. (2016), other processes such as the consumption of NADPH by transhydrogenases may also impart measurable fractionations, and may also vary between aerobes and anaerobes. Reduction of NADP^+^ by still other pathways that are not present in aerobes, such as H_2_-consuming hydrogenase, offer still another possibility.

Table 2 documents the presence or absence of metabolic pathways (bold) and of genes for key enzymes (not bold) involved in NADPH processing, all based on the available annotated genomes of the studied strains. This analysis includes both those genes implicated in NADPH production or fractionation from Zhang et al. (2009) and others such as transhydrogenase variants highlighted in Price et al. (2014). All of the microbes in this study have complete sequenced genomes except *D. hydrogenophilus*, which is necessarily absent from this analysis. One important caveat is that we are looking at genome content and not gene expression, thus differences in expressed metabolic capacity under varying conditions cannot be assessed.

**Table 2:** **Metabolic pathways and selected gene content of strains**.

	**EC**	**Gene**	***E. coli***	***P. denitrificans***	***S. oneidensis***	***D. autotrophicum***	***D. propionicus***	***D. multivorans***	***D. alaskensis***
**Glycolysis**			**+**	**+**	**+**	**+**	**+**	**+**	**−**
Glyceraldehyde-3P dehydrogenase	1.2.1.12	gapA	+	+	+	+	+	+	+
**Oxidative PP pathway**			**+**	**+**	**+**	**−**	**−**	**−**	**+**
Glucose-6-dehydrogenase	1.1.1.49	zwf	+	+	+	−	+	−	+
6-phosphogluconate dehydrogenase	1.1.1.44	gntZ	+	+	+	−	+	−	+
**Non-oxidative PP pathway**			**+**	**+**	**+**	**+**	**+**	**+**	**+**
**TCA cycle**			**+**	**+**	**+**	**−**	**−**	**+**	**−**
Citrate synthase	2.3.3.1	cs	+	+	+	+	+	+	−
Aconitase	4.2.1.3	acnA	+	+	+	+	+	+	+
Isocitrate dehydrogenase	1.1.1.42	idh	+	+	+	+	+	+	−
2-oxoglutarate dehydrogenase	1.2.4.2	sucA	+	+	+	−	−	+	−
Succinate dehydrogenase	1.3.5.1	sdhA	+	+	+	−	+	+	−
Malate dehydrogenase	1.1.1.37	mdh	+	+	+	−	+	+	−
Pyruvate dehydrogenase	1.2.4.1	Pdh	+	+	+	+	+	+	−
**Reductive Acetyl-CoA**			**−**	**+**	**−**	**+**	**−**	**+**	**+**
Formate dehydrogenase	1.2.1.43	fdhAB	−	+	−	+	−	+	+
Bifunctional 5,10-methylene-tetrahydrofolate dehydrogenase	3.5.4.9	folD	+	+	+	+	+	+	+
5,10-methylenetetrahydrofolate reductase	1.5.1.20	metF	+	+	+	+	+	+	+
**Serine pathway**			−	+	−	+	−	−	+
Hydroxypyruvate reductase	1.1.1.81	ttuD	−	+	−	+	−	+	+
Malate dehydrogenase	1.1.1.37	mdh	+	+	+	−	+	+	−
**Reductive monocarboxylic cycle**			**+**	**−**	**−**	**+**	**−**	**−**	**+**
Formate C-acyltransferase	2.3.1.54	pflD	+	−	+	+	−	−	+
Pyruvate ferredoxin/flavodoxin oxidoreductase	1.2.7.1	por/nifJ	+	−	−	+	+	+	+
**Transhydrogenases**			**+**	**+**	**+**	**+**	**+**	**+**	**+**
membrane-bound	1.6.1.2	pntAB	+	+	+	+	−	+	−
Soluble	1.6.1.1	udhA	+	−	−	−	−	−	−
electron-bifurcating	1.4.1.13	nfnAB	−	−	−	+	+	+	+
**Others**									
ferredoxin NADP reductase (fpr)	1.18.1.2	fpr	+	+	+	+	−	+	+
NADP+ → NAD+ Kinase (nadK)	2.7.1.23	nadK	+	+	+	+	+	+	+

At the broadest level, the pangenome of the studied strains is extremely variable with only a limited number of relevant genes—and no complete pathways—present in all strains. The main exception to this pattern is the non-oxidative branch of the pentose phosphate pathway, which is present in all strains but does not produce NADPH. Major producers of NADPH during glucose metabolism in *E. coli* are isocitrate dehydrogenase (20–25%), membrane-bound transhydrogenase (35–45%), and the oxidative pentose phosphate pathway (PP pathway; 35–45%; Sauer et al., 2003). Isocitrate dehydrogenase is present in all strains but *D. alaskensis*, but is thought to produce minimal hydrogen isotope fractionation in product NADPH because the reaction is fully committed and acts on a lone substrate hydrogen (Zhang et al., 2009). All three facultative anaerobes possess both the PntAB transhydrogenase as well as a complete PP pathway.

In contrast, only two of the five SRBs (*D. propionicus* and *D. alaskensis*) contain NADP^+^-reducing enzymes of the PP pathway, although all sulfate reducers have the NfnAB electron-bifurcating transhydrogenase. *D. autotrophicum* and *D. multivorans* also contain genes coding for the PntAB transhydrogenase. However, these different enzymatic compliments do not correspond to differences in isotope fractionation by the different SRBs, as the ranges of δ^2^H-values for the most metabolically similar organisms are essentially non-overlapping. The electron-bifurcating transhydrogenase NfnAB that is shown to be relevant to fractionations in *D. alaskensis* by Leavitt et al. (2016) is present all SRBs and so may also play a role here. However, strains with the same transhydrogenase gene content growing on the same substrates express different fractionations, complicating this correspondence.

The most extreme fractionations we observe for SRBs are the lipid ^2^H-depletions produced by *D. propionicus* when grown on any heterotrophic substrate. Notably this organism lacks a complete TCA-cycle and the PntAB transhydrogenase. The lack of a complete TCA-cycle is shared by *D. autotrophicum*, the SRB with the second most depleted lipids, which instead processes heterotrophic substrates through the reductive monocarboxylic pathway (Wood-Ljungahl Pathway; Strittmatter et al., 2009). Moreover, growth on substrates that activate the TCA cycle (acetate, succinate, malate) are broadly associated with ^2^H enrichments of lipids. While it is tempting to conclude that deficiency in TCA cycle genes leads to the observed isotopic depletions, the trend does not hold for *D. alaskensis*, which has a similar gene complement to *D. autotrophicum* but more ^2^H-enriched lipids. It remains possible that lack of TCA-cycle metabolism is broadly associated with ^2^H-depletions, but that *D. alaskensis* is unusually ^2^H-enriched for other reasons, such as operation of the NfnAB transhydrogenase (Leavitt et al., 2016).

In summary, the presence or absence of any single pathway or enzyme does not account for the observed range and distribution of isotope fractionations across the studied strains. There are, however, some correlations, suggesting that biochemical pathways exert partial control. Genes and pathways that we hypothesize to have some effect, but require further testing beyond the scope of this work include 2-oxoglutarate dehydrogenase, the oxidative PP pathway, and the transhydrogenase PntAB. The transhydrogenase NfnAB has been shown independently to have some effect (Leavitt et al., 2016).

### Growth rate

**Figure 3:**
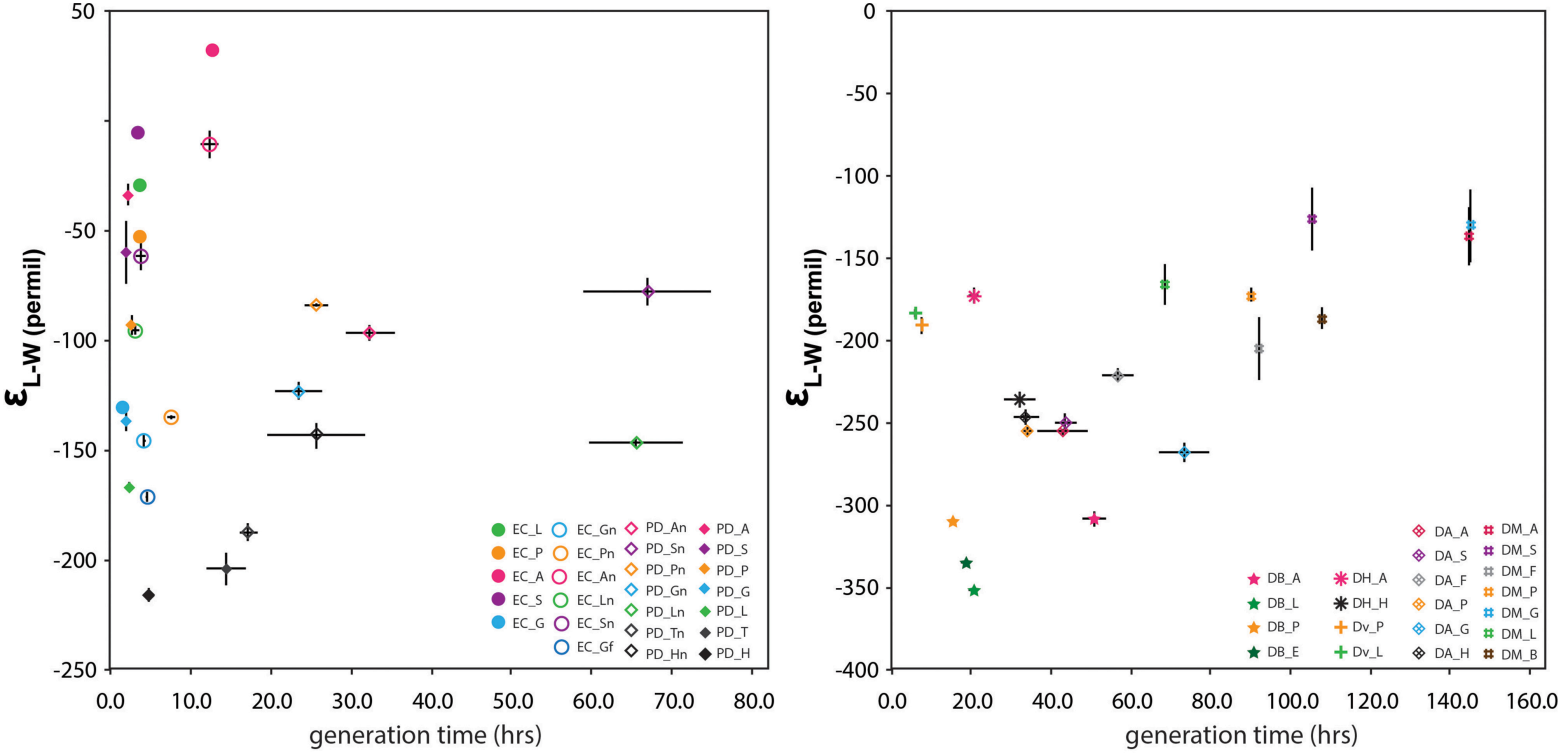
**The relationship between generation time (h) and ε_**L–W**_ for facultative anaerobes (left) and sulfate-reducers (right)**. Error bars for fractionation reflect replicate measurements whereas those for generation time derive from standard error on growth curve regressions.

Growth rate is a potential physiological control on the hydrogen isotopic composition of lipids (Sachse et al., 2012). Working with transhydrogenase mutants of *D. alaskensis*, Leavitt et al. (2016) have shown that hydrogen isotope fractionation is strongly correlated with growth rate in that organism. Growth rate has the potential to impact the δ^2^H-value of the intracellular water pool, the availability of NADPH for anabolism, and cellular equilibration time of any other intracellular pool of hydrogen. It is also a variable that will plausibly change for facultative anaerobes going from oxic to anoxic conditions. To evaluate whether growth rate was a significant factor in our dataset we monitored the growth rate of each strain under each culture condition (Figure 3 and Figure S2). We hypothesized that a slower growth rate (i.e., longer generation time) would increase the extent of H-isotopic exchange between water and NADPH, decreasing observed fractionations in lipids and biomass, and producing a strongly positive slope on Figure 3.

Generation time varied considerably between our cultures ranging from 1.6 h to just over 6 days, with aerobic heterotrophs growing the fastest and *D. multivorans* the slowest (Table 1, Figure 3). Generation time is not correlated with isotopic fractionation (*R*^2^ = 0.03, *N* = 46) and was not shown to be significant on the whole nor for individual metabolic groups via MANOVA analysis. Cultures that produced both the most ^2^H-enriched and ^2^H-depleted lipids have relatively fast generation times (<24 h). Linear regression of isotopic fractionations against growth rates for aerobic, nitrate-respiring, and sulfate-reducing cultures considered separately reveal possible differences between these groups. While the δ^2^H-values of lipids from factitive anaerobes are not correlated with generation time under either condition (*R*^2^ = 0.013 and *N* = 12 for O_2_, 0.02 for NO_3_ and *N* = 13), lipids from sulfate reducing cultures are positively correlated with generation time (0.96‰/h, *R*^2^ = 0.38, *N* = 21). This latter result has the same sign but half the magnitude and with a very different intercept than the correlation identified by Leavitt et al. (2016). In our data, this correlation is driven mainly by the relative ^2^H-depletion and rapid growth of *D. propionicus* and the ^2^H-enrichment and slow growth of *D. multivorans*. For example, if *D. multivorans* is excluded from the regression, this trend disappears (slope –0.38, *R*^2^ 0.01, *N* = 14). It should also be noted that a correlation is not observed for data from individual strains, which might be expected if growth rate was a dominant controlling variable for lipid δ^2^H. Also, MANOVA tests for just SRBs found generation time to be insignificant for describing variations in fractionation. Given that we did not grow *D. alaskensis* under conditions leading to widely varying growth rate, we are unable to directly determine whether our results are compatible with those of Leavitt et al. (2016). Nevertheless, our conclusion is generally opposite theirs, that growth rate is not strongly correlated with δ^2^H across the SRB. Additional experiments under continuous culture conditions might establish a more plausible link between isotopic fractionation and growth rate for the sulfate reducing bacteria.

### Other possible controls on fractionation

**Figure 4:**
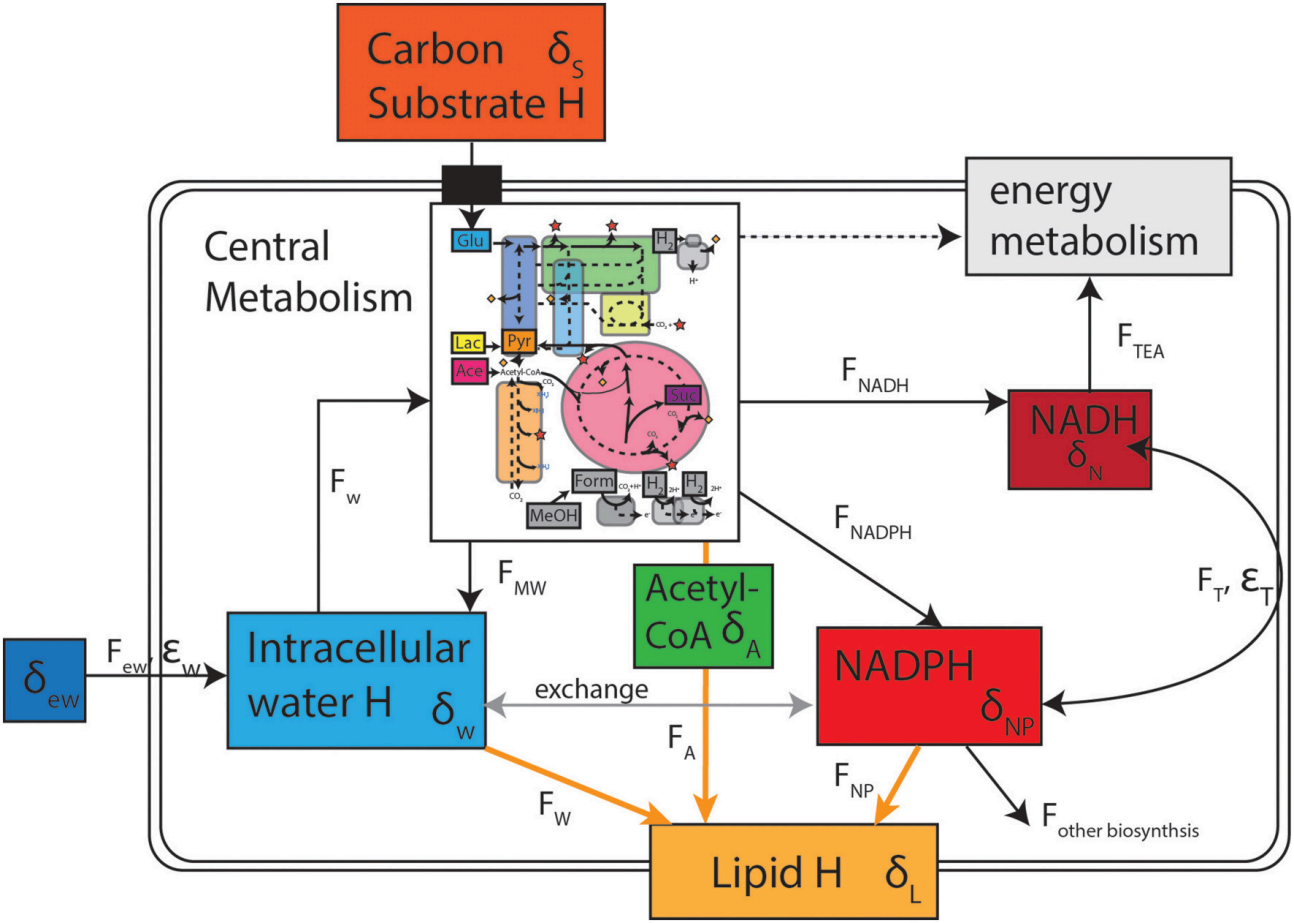
**Conceptual model of the reservoirs of hydrogen as well as fluxes and fractionations that contribute to the ultimate isotopic composition of lipids**. Inset of central metabolism is after that of Sauer et al. (2003) and Zhang et al. (2009).

Based on our understanding of cellular hydrogen fluxes and fractionations (Figure 4), a number of other possible controls on lipid hydrogen isotopes can be considered. Fatty acid biosynthesis is conserved throughout the bacterial domain (White, 2000), so changes in the biosynthetic enzymes are not therefore expected to be a major source of isotopic variability. During biosynthesis, chain elongation occurs by progressive addition of acetyl-CoA molecules to the primer molecule. A number of hydrogen additions occur during hydration of double bonds with either water or NADPH serving as the H donor (White et al., 2005). It follows then that sources of hydrogen on the final fatty acid are acetyl-CoA, water, and NADPH (Figure 4, **F_W_, F_A_, F_NP_**). The stoichiometry of these contributions depends slightly on the chain length and structure of the final lipid (e.g., unsaturated, saturated, or methyl branched), but essentially half of non-exchangeable hydrogen on fatty acids comes from NADPH (Sessions et al., 1999). The other half is sourced equally between intracellular water and the precursor acetyl-CoA. If we assume that the intrinsic biosynthetic fractionations are invariant in a single organism when growing on different substrates, then the isotopic composition of either water or the biosynthetic precursors acetyl-CoA or NADPH must change. In the following sections we explore the potential for intracellular water (δ_w_), transhydrogenase activity (ε_T_), and NADPH residence time and flux effects to affect major changes in lipid isotopic composition (δ_L_).

#### Intracellular water

Intracellular water directly contributes (as H^+^) at least 25% of the carbon-bound hydrogen on a fatty acid. The isotopic composition of this water (δ_w_) is often assumed to be equal to or in static equilibrium with extracellular water (δ_ew_) due to the rapid movement of water in and out of cells through aquaporin channels (Kreuzer-Martin et al., 2006). This assumption is challenged for high-salinity environments where restricted membrane permeability may contribute to the trend of decreasing lipid-water fractionation (Sachse and Sachs, 2008). Intracellular water also derives from metabolic processes (F_MW_), mainly respiration, and the balance between this source and inward diffusion of extracellular water has been shown to change with growth rate and phase (Kreuzer-Martin et al., 2006; Romero-Viana et al., 2013). Kreuzer-Martin et al. (2005, 2006) quantitatively evaluated the sources of intracellular water in *E. coli* and found that during very rapid growth (doubling time of ~0.5 h), a majority of intracellular water was derived from metabolic rather than medium water. This respiratory contribution was reduced during stationary phase and at slower growth rates, thus introducing a mechanism for growth phase related hydrogen isotope effects (Kreuzer-Martin et al., 2005, 2006). Although our cultures were all harvested at a similar growth phase, it is possible that the relative offset between intra- and extra-cellular water could vary between aerobic and anaerobic growth of a single strain or between strains on different substrates. Testing this hypothesis will require measurements of δ_w_ from cultures, which produce different δ_L._ Notably, our cultures were growing more slowly than those shown to produce this effect in Kreuzer-Martin et al. (2006) and we observed similar fractionations for cultures harvested intentionally in stationary phase (see Supplementary data PD_L(SP)_-82), neither of which support a role for changes in metabolic water to lipid δ^2^H.

**Figure 5:**
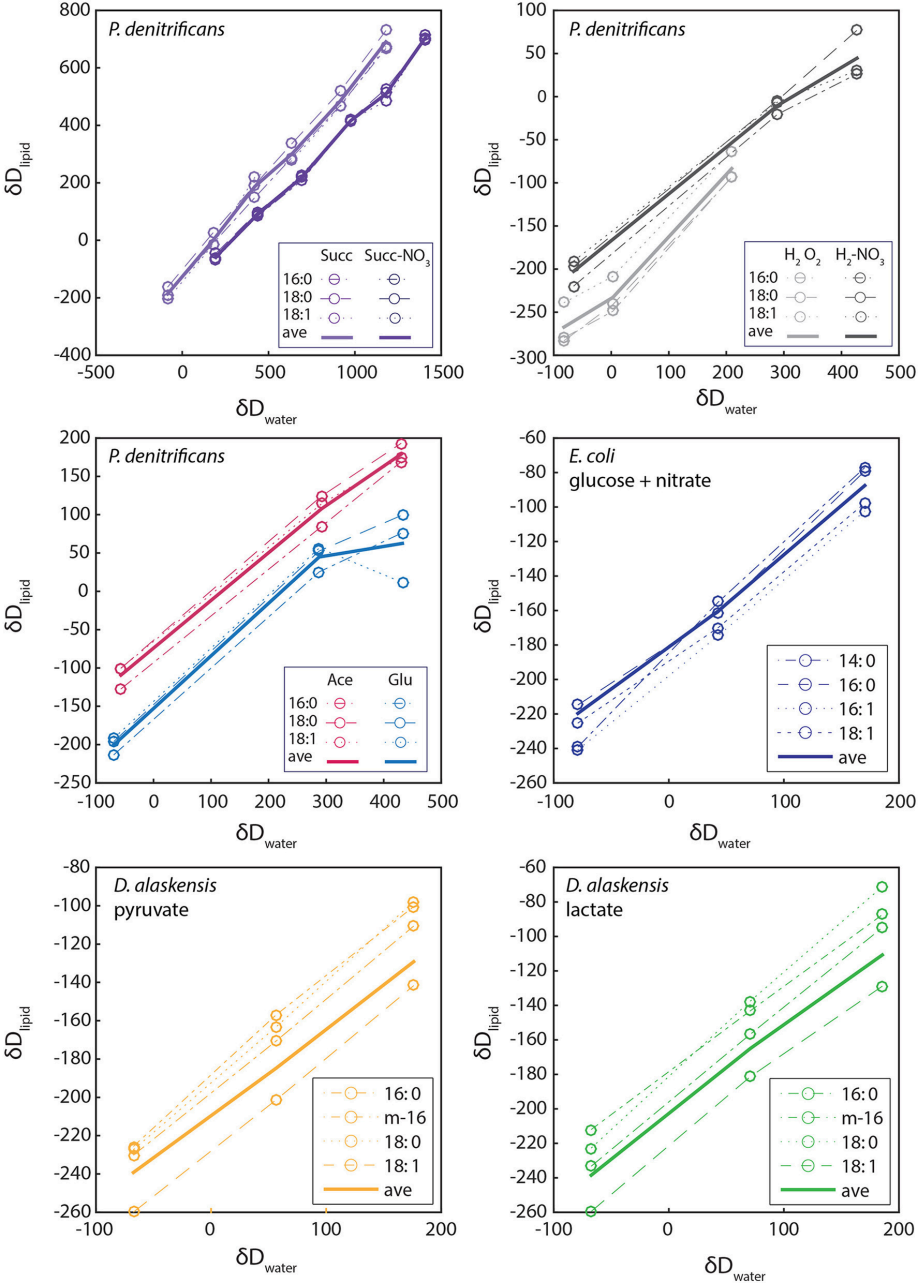
**The relationship between hydrogen isotopic composition of growth water and lipids for ***P. denitrificans, E. coli***, and ***D. alaskensis*****. Linear regressions (not shown) were performed on the points for weighted average lipid values (bold lines), rather than data for individual lipids (dotted and dashed lines, see legend).

We tested extracellular water incorporation in growth trials with *P. denitrificans* and *D. alaskensis* using variable δ^2^H_ew_ and a range of metabolic substrates. For these experiments lipid δ^2^H is strongly correlated with growth medium water δ^2^H in all cases with regression slopes ranging from 0.48 to 0.68 (Figure 5). The slope of linear regressions between δ^2^H_water_ and δ^2^H_lipid_ in Figure 5 are driven by a combination of fractional water incorporation (X_w_), fractionation between lipid and water (ε_l∕w_), and fractionation between lipid and substrate (ε_l∕s_) (See Sessions and Hayes, 2005; Zhang et al., 2009 for discussion). This complexity prevents the direct interpretation of the correlation slope as fractional water incorporation, but comparisons can be made following the logic discussed in Zhang et al. (2009) (Figure S3).

We directly compared δ^2^H of lipids produced during aerobic and anaerobic growth of *P. denitrificans* on succinate and hydrogen for a large range of δ^2^H_water_. We observe a decrease in slope for the anaerobic condition for both substrates, although with an opposite trend in intercept values (Figure 5). Assuming biosynthetic fractionations remain constant, this change in slope could be interpreted as a decrease in fractional water incorporation (or reduced equilibration of other pools with water) under the anaerobic condition.

An alternative interpretation is an increase in isotope fractionation between lipid and substrate. Slopes were identical and intermediate in value for growth on acetate and glucose. We do not see significant change in slope between facultative anaerobes and SRBs with similar results from *D. alaskensis* cultures grown on lactate and pyruvate (slopes 0.48 – 0.60). Our results are entirely within the range of those from Zhang et al. (2009) precluding an obvious mechanistic link between growth water incorporation and the unusual fractionation patterns observed in anaerobes. This approach may however yield more conclusive results with testing of additional substrates and culture conditions.

#### Transhydrogenase activity

Transhydrogenase enzymes moderate the balance between NADPH and NADH in the cell (Griffiths and Roberton, 1966; White, 2000), vary in their activity based on metabolic conditions (Sauer et al., 2003), and are associated with large isotope effects (Jackson et al., 1999). There is thus ample reason to think these enzymes are key determinants in large hydrogen isotopic shifts. Different central metabolic pathways produce varying amounts of NADH vs. NADPH, leading to a potential imbalance between supply and demand of NADPH for anabolism. Transhydrogenase enzymes modulate the transfer of a hydride (H^−^) moiety from NADH to NADP^+^, or from NADPH to NAD^+^. In *E. coli* these two processes are accomplished via two different transhydrogenase enzymes: an energy-dependent, membrane-bound version (PntAB, Table 2) that produces NADPH, and a soluble version (UdhA) that catalyzes the opposite reaction. Isotope labeling experiments in *E. coli* show that growth on glucose results in 35–45% of the NADPH pool being produced via the PntAB transhydrogenase, i.e. NADPH is underproduced relative to anabolic demand. In contrast, overproduction of NADPH is observed during growth on acetate, and this surplus is reduced using the soluble UdhA enzyme (Sauer et al., 2003). Given a normal isotope effect for both classes of enzymes (typically 800–3500‰; Jackson et al., 1999), we might expect that underproduction of NADPH leads to ^2^H-depletion (NADPH is the enzymatic product) and overproduction leads to ^2^H-enrichment (NADPH is the reactant), a pattern that is broadly similar to that observed in *E. coli*.

In addition to these two transhydrogenases found in *E. coli*, a third, electron bifurcating transhydrogenase (NfnAB) has been shown to be important in the growth of *D. alaskensis* (Price et al., 2014). This enzyme is reversible depending on substrate and allows for ATP production despite the high activation energy of sulfate (Price et al., 2014). Mutants deficient in NfnAB are shown to produce lipids that are ^2^H-enriched by up to 75‰relative to the wild type under conditions of NADPH consumption, whereas they are nearly identical under conditions of NADPH production by NfnAB (Leavitt et al., 2016). This is the opposite direction of fractionation than predicted by a normal isotope effect, i.e. consumption of NADPH by NfnAB should lead to ^2^H enrichment, and eliminating that activity should correspond to ^2^H depletion.

The strains studied here are all genetically capable of expressing at least one transhydrogenase variant with the exception of *D. propionicus* (Table 2), although they differ significantly in how many and which versions they possess. *E. coli* is the only organism to possess genes for both the membrane-bound (PntAB) and the soluble (UdhA) transhydrogenases as described in Sauer et al. (2003). *P. denitrificans, S. oneidensis*, and *D. multivorans* contain only PntAB coding genes, *D. alaskensis* has only those for NfnAB, whereas *D. autotrophicum* contains genes for both PntAB and NfnAB. While genetic potential does not necessarily indicate enzymatic activity, the inverse is true: organisms lacking a specific gene lack the corresponding transhydrogenase. For instance, expression of transhydrogenases by *E. coli* and *P. denitrificans* when switching between growth on glucose to acetate, must vary as *E. coli* has been demonstrated to upregulate the expression of the UdhA encoding transhydrogenase that *P. denitrificans* lacks (Sauer et al., 2003). Interestingly, we do observe large ^2^H-enrichment of *E. coli* lipids relative to those of *P. denitrificans* when growing on acetate but not on glucose, consistent with this prediction (Figure 1). Direct demonstration of differential transhydrogenase expression between these cultures and conditions will be required to prove a mechanistic link.

Some SRBs in this study have the genes for both PntAB and NfnAB, some have only PntAB, and still others have neither. However, the hydrogen isotope fractionations by SRBs we measured do not support significant differences in transhydrogenase fractionations. *D. multivorans* and *D. alaskensis* produce similar, small ranges of fractionations despite having genes for PntAB and NfnAB, respectively. *D. autotrophicum* has strongly ^2^H-depleted lipids with little variation between growth substrates, despite having the genetic potential to produce both pntAB and nfnAB. The strong ^2^H-depletion observed for *D. propionicus* despite a lack of transhydrogenase genes is interesting, but inconclusive at this point. In summary, the potential for transhydrogenase enzymes to influence the isotopic composition of NADPH (δ_NP_) and thus lipids is clear, and there is experimental evidence that this is so in some particular organisms. However, it appears unlikely to be a single, over-riding control that could explain all hydrogen isotope variations for all of the studied organisms.

#### NADPH isotope exchange and cellular flux effects

NADPH contributes up to half of the carbon-bound hydrogen on fatty acids and thus has significant leverage to influence lipid isotopic composition. Variation in the isotopic composition of the H^−^ donated by NADPH (δ_NP_) is hypothesized to be the dominant mechanism by which central metabolism influences δ_L_(Zhang et al., 2009; Dirghangi and Pagani, 2013a,b; Heinzelmann et al., 2015b). Previously, control of δ_NP_ has been ascribed mainly to NADPH-reducing pathways (“supply”), although both the demand for NADPH and the process of isotopic exchange could also influence its δ^2^H-value. The latter, in particular, might vary with the cellular residence time of reduced NADPH, which in turn might plausibly vary between aerobic/anaerobic metabolism.

It is well-established that the cellular quotient of NADP(H) varies between strains and across growth conditions (London and Knight, 1966; Wimpenny and Firth, 1972; Matin and Gottschal, 1976; Karl, 1980). London and Knight (1966) specifically noted differences in the relative abundance of NAD^+^ between strict anaerobes, facultative anaerobes, and aerobes. Focusing just on NADPH, if we assume that a larger cellular quotient of reduced NADPH is correlated with a longer residence time for each molecule, then greater hydrogen isotope exchange with cellular water could result in damping the isotopic signals associated with NADPH supply. Existing reports are conflicting about the precise rate of isotopic exchange of NADPH *in vivo*, but at least one study (Saito et al., 1980) has observed rapid exchange *in vitro*. The extent to which either the size or the oxidation state of the NADPH reservoir influences lipid δ^2^H is not known, but this appears to be a promising avenue for further investigation.

It also likely that the various downstream biosynthetic processes that constitute the demand for reduced NADPH fractionate hydrogen isotopes. Even if individual isotope biosynthetic effects remain constant, a change in the relative proportions of downstream products (e.g. more or less lipids relative to other biochemical pools) could change their individual isotopic compositions even while the bulk isotopic composition changes very little (Hayes, 2001). Comparison of the bulk isotopic data to that of fatty acids (Figure 2A) does not favor this hypothesis. The δ^2^H-values of both lipids and bulk biomass change with growth substrate, and—more importantly—the order of relative ^2^H-depletion reverses (e.g., lipids are ^2^H-depleted relative to biomass in some cultures, and ^2^H-enriched in others). The latter pattern could not arise simply from changing the relative proportions of anabolic products with constant isotope effects.

Instead, the co-varying isotopic compositions of bulk biomass and lipids are more consistent with them receiving different amounts of H from a common source, whose δ^2^H-value varies between experiments. For example, our data (Figure 2A) can be reasonably fit by a 2-component mixing model, with one component (presumably water + substrate) having a constant δ^2^H of −64‰ and the other (presumably NADPH) having δ^2^H that varies between 0 and −700‰, with 50% of lipid hydrogen and only 13.5% of bulk biomass hydrogen coming from NADPH (Figure S4). This simple model is certainly an oversimplification, and is not a unique solution, but is at least consistent with what is known about the relative contributions of NADPH to lipids vs. other biomolecules such as proteins and sugars.

## Conclusions

Our study reveals additional complexity of hydrogen isotope fractionation in microbial species. Two distinct patterns of fractionation were measured among heterotrophic bacteria: one in facultative nitrate-reducers that is qualitatively similar to that seen previously in aerobes, and a second in sulfate-reducing bacteria that is entirely distinct. Our data confirms that growth substrate is the dominant controlling variable for lipid δ^2^H-values of facultative anaerobes, consistent with previous reports. In contrast, SRB produce ^2^H-depleted lipids, apparently regardless of metabolic pathway or substrate, and instead converge on strain-specific values. Generally speaking, lipids produced during nitrate reduction and aerobic respiration tend to be more enriched, whereas those produced during sulfate reduction tend to be more depleted and in a very similar range as photoautotrophy. The distinction between autotrophic and heterotrophic growth observed by Zhang et al. (2009) and emphasized subsequently (Valentine, 2009; Heinzelmann et al., 2015b), was replicated in nitrate reducers but not sulfate reducers. In SRB, utilization of heterotrophic substrates results in the production of lipids that match or exceed ^2^H-depletions observed for autotrophic growth. Whether this result represents an exception or the rule for other obligate anaerobes is still unknown.

## Author contributions

This manuscript is part of the thesis work of MO who completed the bulk of the experiments, measurements, and writing of this manuscript. KD cultured one strain of bacteria and contributed significantly to the genetic components of the manuscript. MF contributed measurements of bulk microbial biomass that significantly shaped the arguments within the manuscript. The majority of experiments and measurements were completed in the laboratories of AS who also contributed significantly to the intellectual development of the project, advisement of MO, and formulation of the manuscript. All authors contributed to writing and editing of the manuscript.

### Conflict of interest statement

The authors declare that the research was conducted in the absence of any commercial or financial relationships that could be construed as a potential conflict of interest.
