# Supplementary material for: Fractionation of Hydrogen Isotopes by Sulfate- and Nitrate-Reducing Bacteria
Source: Front Microbiol. 2016 Aug 2;7:1166. doi: 10.3389/fmicb.2016.01166 (PMC4969296; doi:10.3389/fmicb.2016.01166)
Supplement: Supplementary file 2 [file Image1.PDF]

Supplementary Figures

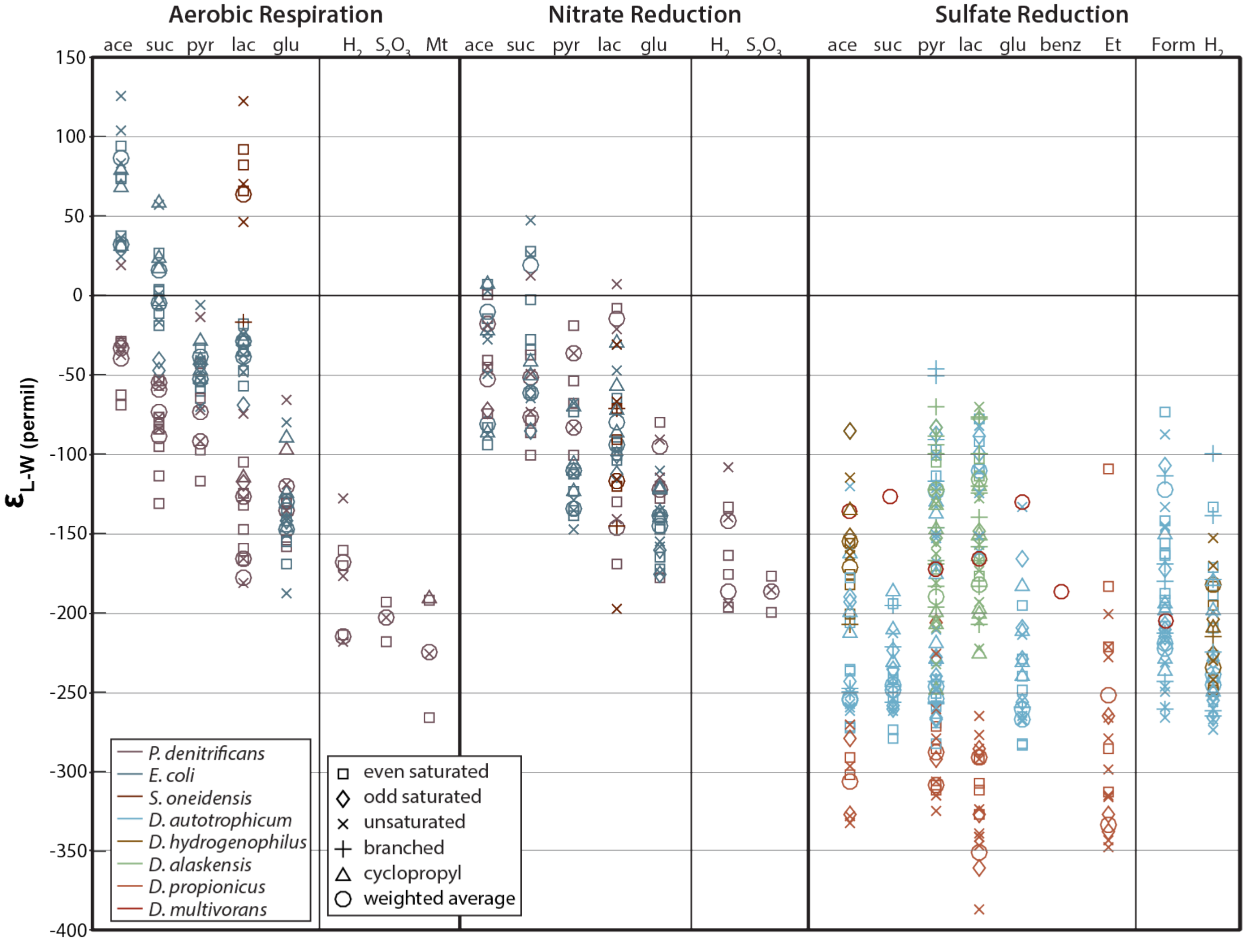

Figure S1: Compound-specific $\varepsilon_{L\text{-}W}$ grouped by metabolism. Colors indicate the strain, symbols indicate the fatty acid structural variety, and x-axis position indicate metabolism as labeled.

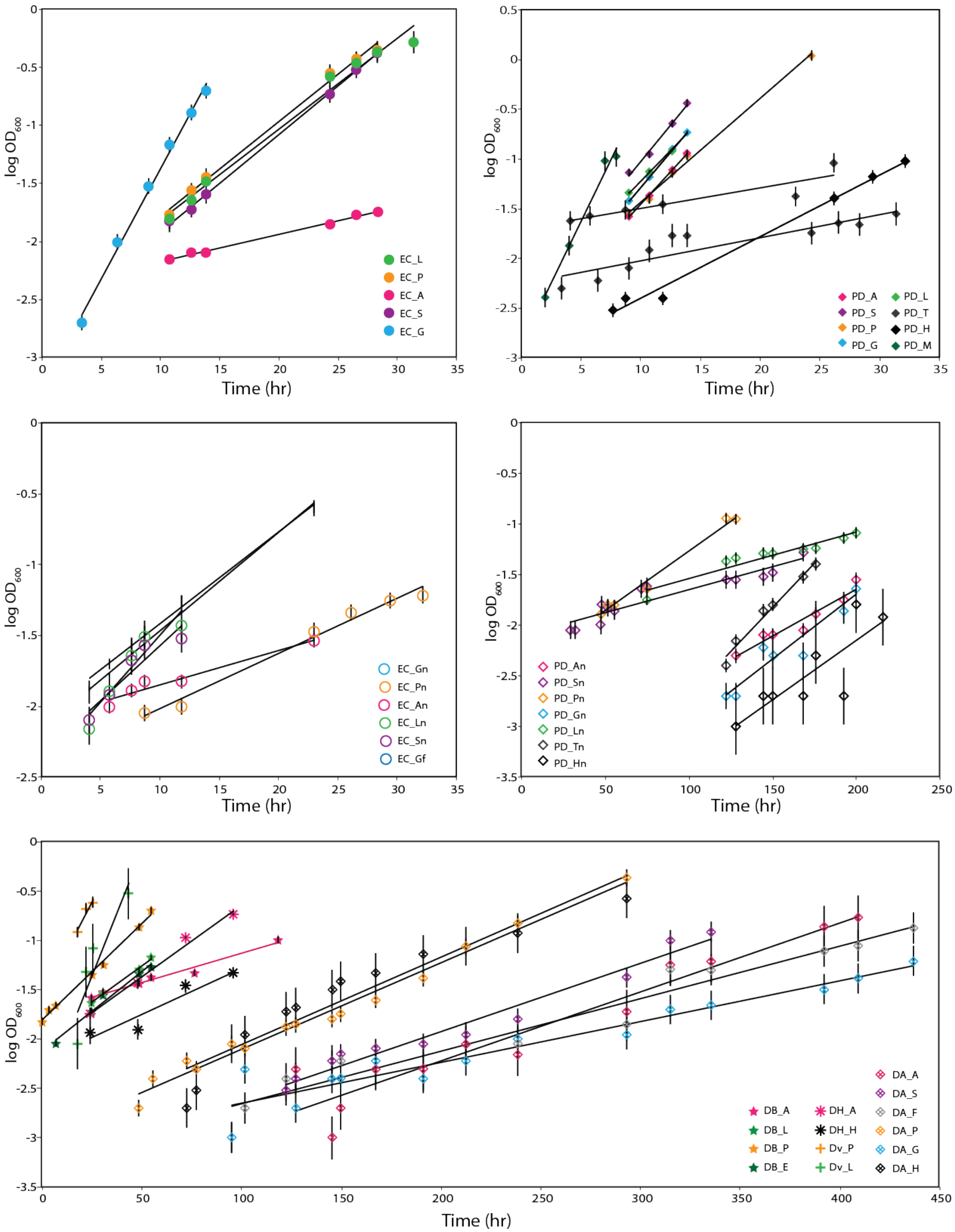

Figure S2: Growth curves for aerobic (top), nitrate reducing (middle), and sulfate reducing (bottom) cultures. Only the exponential phase is plotted. Generation times were calculated from the slopes of regression lines after Widdle, 2010. Error bars are estimated based on the standard error of regressions.

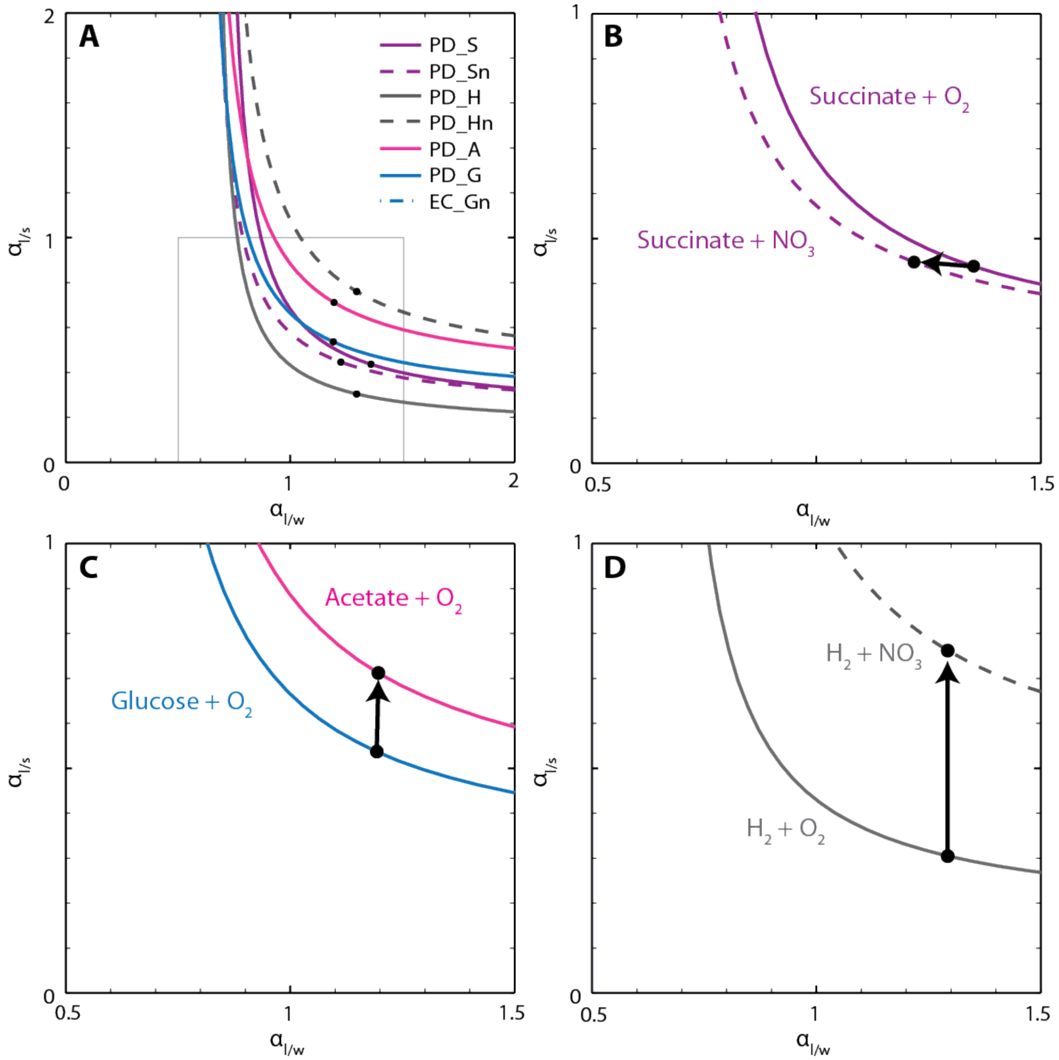

Figure S3: Fractionation factor curves for weighted average lipids of *P. denitrificans* growing on a range of metabolic substrates under aerobic and anaerobic conditions. Circles illustrate the point on the curve where $X_W = 0.5$

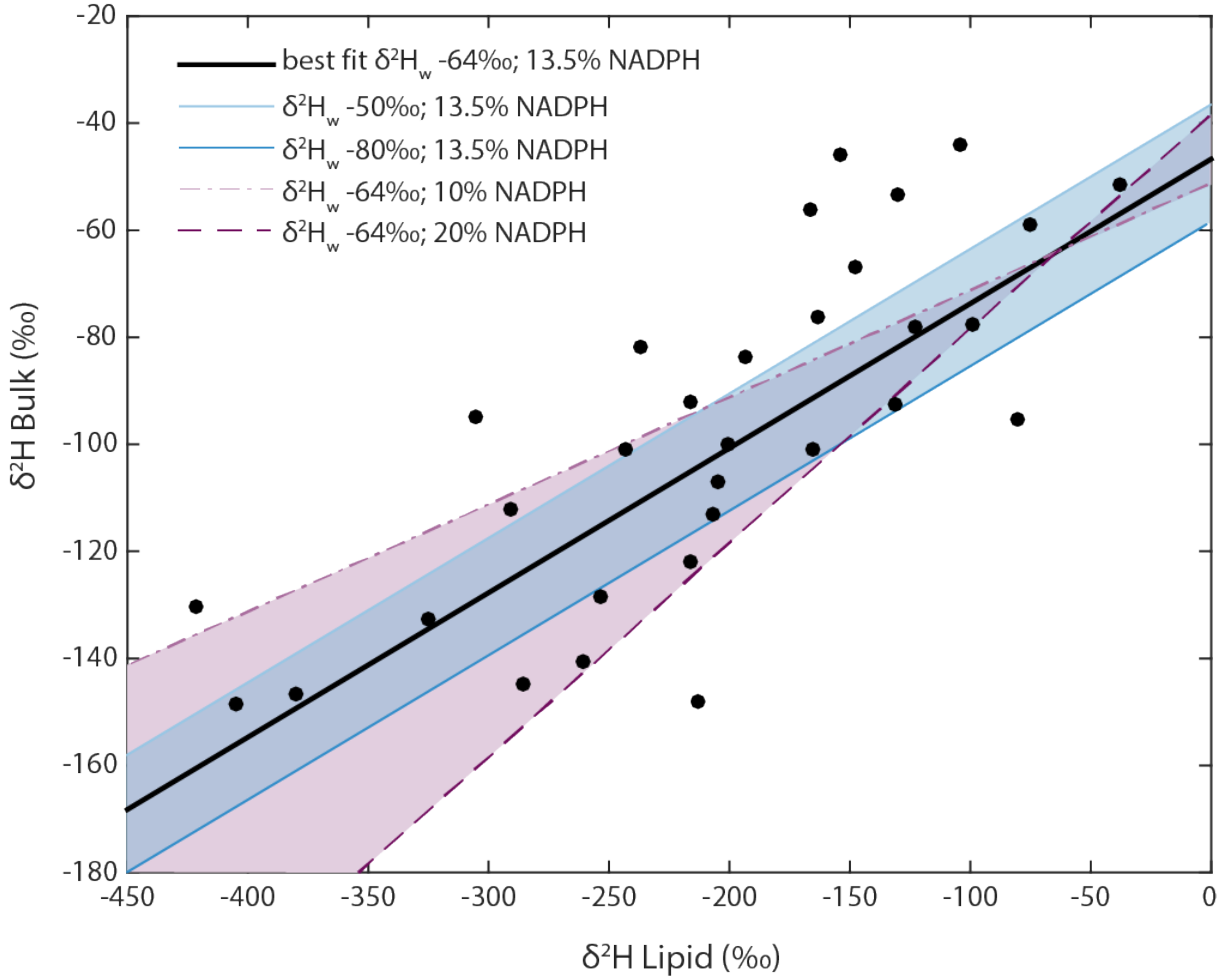

Figure S4: Results of a two component mixing model with one component (representing water + substrate) having a constant δ^2H of -64‰ and the other (NADPH) having δ^2H that varies between 0 and -700‰, with 50% of lipid hydrogen and only 13.5% of bulk biomass hydrogen coming from NADPH (black line). Data from this study is shown in black circles. Sensitivity to the isotopic composition of component 1 ranging from -50 to -80‰ is outlined by blue polygon and sensitivity to the relative contribution of component 2 (NADPH) to bulk lipid ranging from 10 to 20% is shown in the pink polygon.
